# Mobile mammography in New York City: analysis of 32,350 women utilizing a screening mammogram program

**DOI:** 10.1038/s41523-022-00381-6

**Published:** 2022-01-21

**Authors:** Astrid Botty van den Bruele, Varadan Sevilimedu, Maxine Jochelson, Silvia Formenti, Larry Norton, Virgilio Sacchini

**Affiliations:** 1 Breast Service, Department of Surgery, Memorial Sloan Kettering Cancer Center, New York, NY USA; 2 Department of Epidemiology & Biostatistics, Memorial Sloan Kettering Cancer Center, New York, NY USA; 3 Department of Radiology, Memorial Sloan Kettering Cancer Center, New York, NY USA; 4 Department of Radiation Oncology, Weill Cornell Medicine, New York, NY USA; 5 Breast Medicine, Department of Medical Oncology, Memorial Sloan Kettering Cancer Center, New York, NY USA

**Keywords:** Breast cancer, Diagnosis, Cancer screening

## Abstract

Mobile mammography vans (mammovans) may help close the gap to access of breast cancer screening by providing resources to underserved communities. Minimal data exists on the populations served, the ability of mammovans to reach underserved populations, and the outcomes of participants. We sought to determine the demographic characteristics, number of breast cancers diagnosed, and number of women who used the American Italian Cancer Foundation (AICF) Mobile, No-Cost Breast Cancer Screening Program within the five boroughs of New York City. Data were collected by the AICF from 2014 to 2019 on a voluntary basis from participants at each screening location. Women aged 40 to 79 years who had not had a mammogram in the previous 12 months were invited to participate. Each participant underwent a clinical breast exam by a nurse practitioner followed by a screening mammogram. Images were read by a board-certified radiologist contracted by the AICF from Multi Diagnostic Services. There were 32,350 participants in this study. Sixty-three percent reported an annual household income ≤$25,000, and 30% did not have health insurance. More than half of participants identified as either African American (28%) or Hispanic (27%). Additional testing was performed for 5359 women found to have abnormal results on screening. In total, 68 cases of breast cancer were detected. Breast cancer disparities are multifactorial, with the greatest factor being limited access to care. Mobile, no-cost mammogram screening programs show great promise in helping to close the gap to screening access.

## Introduction

Breast cancer stage and tumor biology are two of the most important drivers of disease-specific cancer outcomes. Disparities in health care access, or social determinants of health, can have a negative impact on survival but often go underappreciated as a component of care. Multiple studies have attempted to address breast cancer health disparities among women of different races, socioeconomic statuses, and geographic locations. Data consistently demonstrate that the social disparities affecting breast cancer including, but not limited to, low socioeconomic status, lack of health insurance, racial discrimination, and poor social support, are associated with worse breast cancer prognosis^1–3^. Residential segregation and municipal district of residence have also been cited as key factors by which the social environment is theorized to affect health^4–7^, as these are related to health care access and availability of medical services, including screening.

A wealth of literature has demonstrated that regular screening mammography increases the likelihood of avoiding death from breast cancer^8^. The availability of screening centers, a component of access, has a tremendous impact on breast cancer outcomes. Although mammographic capacity in the United States (US) may be adequate on average, the unequal geographical distribution of facilities creates an obstacle for many women and has been identified as an independent predictor of late-stage diagnosis^9–11^. Mobile screening mammography vans, or mammovans, have the unique capability of traveling to traditionally underserved areas. The use of mammovans is theorized to assist in closing the gap to access by bringing this critical resource to the community itself. Despite the existence and increasing use of mammovan outreach in the US, there remains a paucity of data regarding the demographic characteristics and outcomes among patient populations utilizing these services.

With almost 16,000 women diagnosed and more than 2500 women dying of their disease yearly^12^, breast cancer remains one of the most common cancers among women in New York State. Rates of mammography screening tend to be lower among the uninsured and those living in underrepresented areas^13^. Aggressive action has been taken to improve health care services^14^, and, in conjunction with existing governmental programs, several nonprofit organizations have emerged to provide additional aid to communities within New York State. One such organization, the American Italian Cancer Foundation (AICF), has a long tradition of serving economically disadvantaged, medically underserved women through outreach and education^15^. The AICF was cofounded in 1980 by Umberto Veronesi, MD as a method to increase international collaboration in the fight against cancer^15^. As one of the core programs developed by the foundation, the Mobile, No-Cost Breast Cancer Screening Program provides a free screening service to women in need. The program is funded by the New York City Council, New York State Senate, and New York State Assembly, private foundations, and individual donors. The AICF Mobile, No-Cost Breast Cancer Screening Program provides mammography screening vans to the five boroughs of New York City: Queens, Manhattan, Brooklyn, Staten Island, and the Bronx. Each screening event is conducted in partnership with a community-based organization^15^. By emphasizing the importance of early detection, the AICF program offers no-cost breast cancer screening to those who may not otherwise have the financial resources or readily available access to screening facilities.

We sought to determine the number of breast cancer diagnoses and elucidate the demographic information of women using the AICF program within the five boroughs of New York City.

## Results

### Patient demographic characteristics

**Fig. 1:**
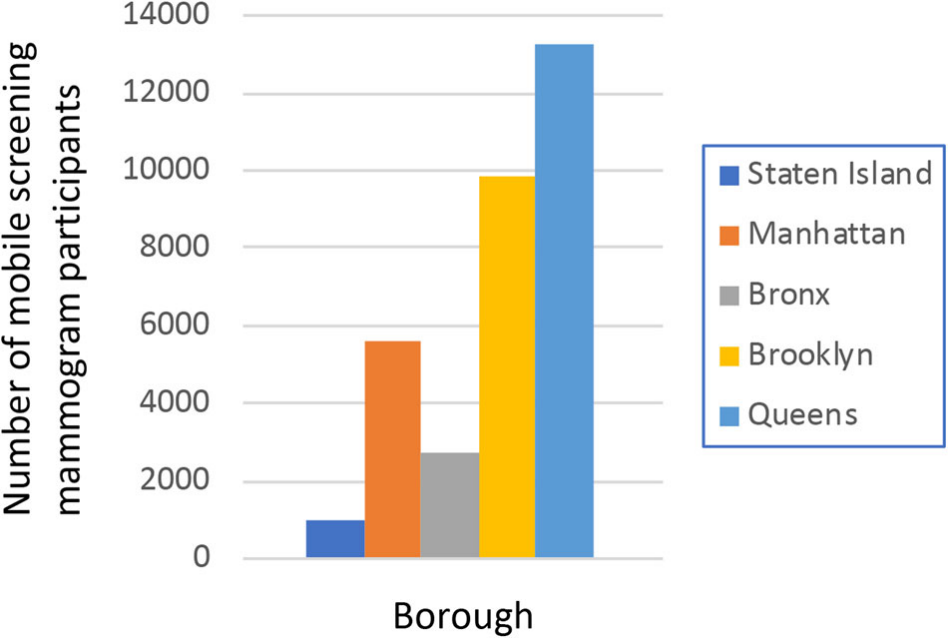
Participants by borough utilizing the Mobile Mammogram Screening Program from 2014 to 2019.

**Fig. 2:**
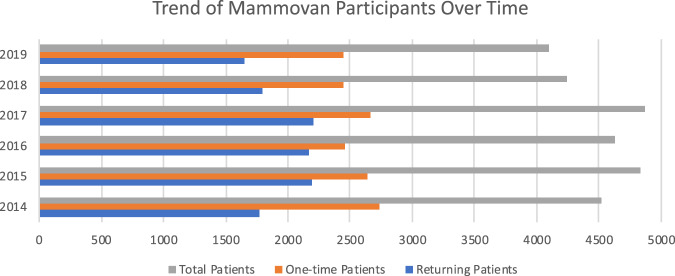
Trends of Mobile-Mammogram utilization over time. These numbers include total participants, one time participants, and returning participants.

There were 32,350 women who participated in the AICF Mobile, No-Cost Breast Cancer Screening Program from 1 January 2014 to 31 December 2019 (Fig. 1). There were over 4000 mammograms performed per year (Fig. 2). Roughly 57% (15378/27169) were one-time participants, and 11,893 (36%) were recorded as returning patients. Demographic information was recorded by borough of screening location (Table 1). Overall, 10,523 women (63%) reported an annual household income ≤$25,000 (Table 1). Of the women who reported their insurance status, 8196 (30%) did not have health insurance (Table 1). Time-trend analysis in insurance coverage revealed that the number of uninsured gradually declined with each year (data not shown).

**Table 1:** Self-reported demographic information by borough of screening location (*N* = 32,350).

Characteristic	Queens	Brooklyn	Bronx	Manhattan	Staten Island	*P* value
Race and ethnicity						<0.001
African American (*N* = 7503)	3144 (28.0)	2283 (28.9)	666 (29.5)	1296 (27.7)	114 (13.9)	–
Asian/Pacific Islander (*N* = 3602)	1951 (17.4)	710 (9.0)	240 (10.6)	575 (12.3)	126 (15.3)	–
Hispanic (*N* = 7268)	3030 (27.0)	1564 (19.8)	834 (36.9)	1549 (33.1)	291 (35.4)	–
Caucasian (*N* = 5466)	1731 (15.4)	2560 (32.4)	225 (10.0)	738 (15.8)	212 (25.8)	–
Native American (*N* = 693)	337 (3.0)	144 (1.8)	73 (3.2)	133 (2.8)	6 (0.73)	–
Other (*N* = 2371)	1052 (9.4)	637 (8.1)	222 (9.8)	387 (8.3)	73 (8.9)	–
Total (*N* = 26,903)	11,245	7898	2260	4678	822	–
Yearly income, $						<0.001
<10,000 (*N* = 3201)	1361 (20.3)	878 (16.6)	272 (20.8)	607 (20.7)	83 (16.6)	–
10,001–15,000 (*N* = 3330)	1389 (20.7)	974 (18.5)	296 (22.7)	575 (19.6)	96 (19.2)	–
15,001–25,000 (*N* = 3992)	1678 (25)	1271 (24.1)	314 (24)	625 (21.3)	104 (20.8)	–
25,001–50,000 (*N* = 3881)	1448 (21.6)	1319 (25)	281 (21.5)	699 (23.7)	134 (26.8)	–
>50,000 (*N* = 2321)	838 (12.5)	834 (15.8)	143 (11)	423 (14.4)	83 (16.6)	–
Total (*N* = 16,725)	6714	5276	1306	2929	500	–
Insurance status						<0.001
Uninsured (*N* = 8196)	3484 (31.5)	2365 (28.5)	713 (31.2)	1392 (29.4)	242 (33.4)	–
Medicare (*N* = 5568)	2140 (19.4)	1869 (22.5)	419 (18.3)	995 (21)	145 (20)	–
Medicaid (*N* = 5382)	2330 (21.2)	1433 (17.3)	517 (22.6)	985 (20.8)	117 (16.2)	–
Private (*N* = 7949)	3102 (28.1)	2625 (31.7)	638 (27.9)	1364 (28.8)	220 (30.4)	–
Total (*N* = 27,095)	11,056	8292	2287	4736	724	–
Age, years						<0.001
<40 (*N* = 2823)	1003 (7.6)	939 (9.6)	231 (8.5)	521 (9.3)	129 (13.2)	–
40–49 (*N* = 8616)	3207 (24.2)	2627 (26.8)	853 (31.5)	1586 (28.3)	343 (35.2)	–
50–59 (*N* = 8181)	3915 (29.6)	2155 (22)	718 (26.5)	1221 (21.8)	172 (17.7)	–
60–69 (*N* = 5466)	2357 (17.8)	1688 (17.2)	348 (12.9)	947 (16.9)	126 (12.9)	–
70–79 (*N* = 1489)	678 (5.1)	487 (5)	73 (2.7)	224 (4)	27 (2.8)	–
≥80 (*N* = 5775)	2074 (15.7)	1923 (19.6)	485 (17.9)	1116 (20)	177 (18.2)	–
Total (*N* = 32,350)	13,234	9819	2708	5615	974	–

Data are no. (%).

**Fig. 3:**
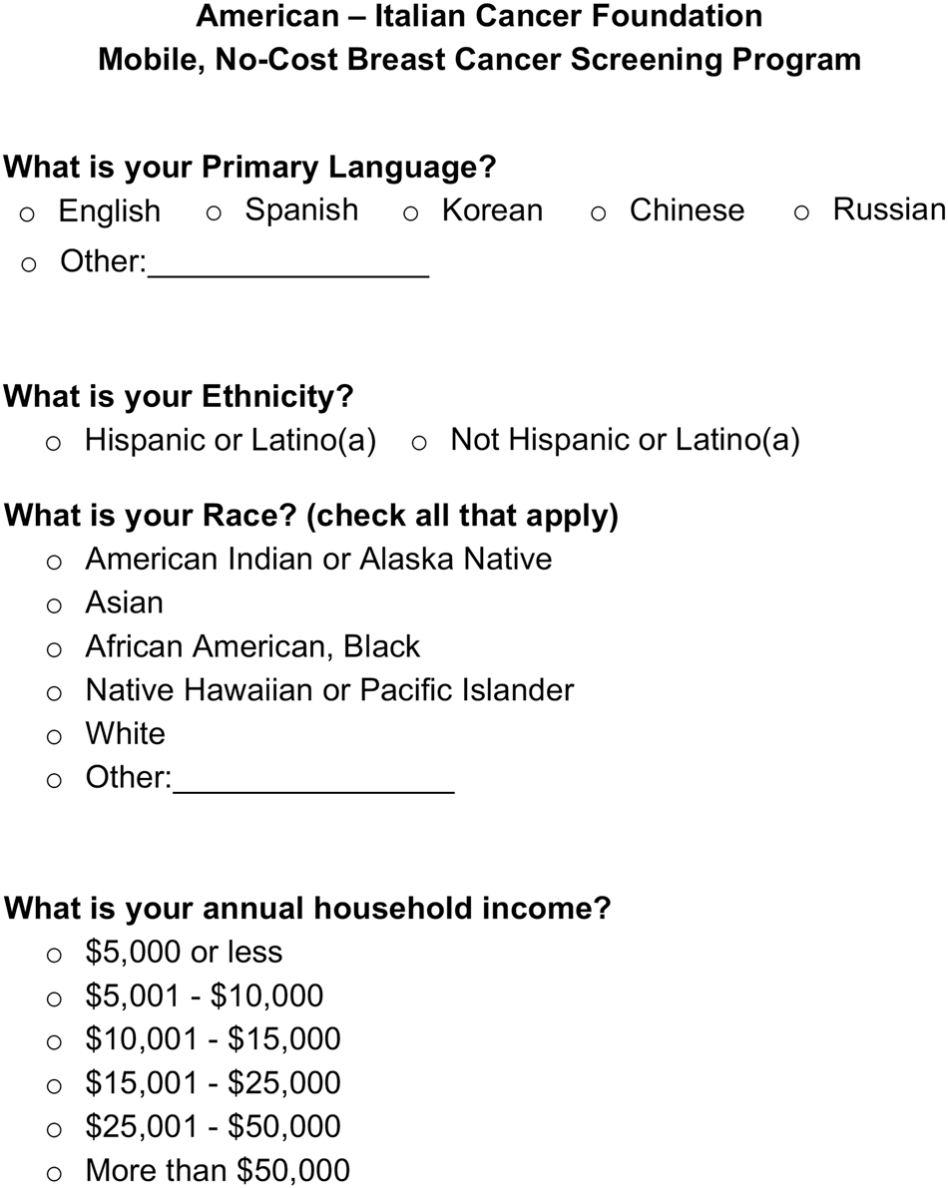
Example of the Participant Questionnaire.

In total, 26,903 participants (83%) reported their race and ethnicity (Fig. 3 and Table 1); 28% identified as African American and 27% identified as Hispanic. Queens, the Bronx, and Manhattan had a similar distribution of African American participants (28–30%). The Bronx, Manhattan, and Staten Island had a dominant mixture of both Caucasian and Hispanic participants (Table 1). In Brooklyn, 32% of participants identified as Caucasian.

Of the participants for whom we have available data, women aged 40–59 years made up 52% of those screened. In Staten Island, 48% of participants were aged <50 years; in the Bronx, 40% were aged <50; and in Queens, ~32% were aged <50. In Queens, Brooklyn, and Manhattan, 18%, 17%, and 17% of participants, respectively, were aged 60–69 years (Table 1). Of the entire cohort, 12% (3740/32350) reported they underwent their first screening mammogram through this program (Table 2). It is uncertain whether the remaining participants underwent follow-up mammograms using this program or whether first-time mammograms were underreported.

**Table 2:** Baseline screening, additional testing, and number of cancers identified per year.

Year	Mammograms performed	Patients undergoing baseline screening	Patients requiring additional testing	Breast cancers diagnoses
2014	4468	581	625	6
2015	4797	635	677	14
2016	4562	577	798	20
2017	4818	672	1011	13
2018	6671	633	1115	8
2019	7072	642	1133	7

### Cancer detection

The AICF assisted in additional testing of 5359 women who were found to have abnormal results on screening mammography. Approximately 14% of those with an abnormal mammogram also had an abnormal CBE. The recall rate per year averaged ~9%. From 2015 to 2019, there were 4719 patients who were recommended for additional follow-up, although 580 (12%) were unable to be contacted or were lost to follow-up (Table 3).

**Table 3:** Patients lost to follow-up per year.

Year^a^	Lost to follow-up	Total followed-up
2015	105	700
2016	108	783
2017	178	1011
2018	85	1115
2019	104	1110

^a^Data for 2014 were not available.

In total, 68 cases of breast cancer were detected. Once diagnosed with breast cancer, these women were then directed to a definitive treatment center and were given additional assistance in making the appropriate follow-up. The AICF program was no longer involved once a secure treatment plan was in place. Data on disease stage, treatment, and/or outcomes were not collected unless the women contacted the program with updates during or after treatment. Of 10 known cancers with follow-up information, 2 were stage 0 (in situ disease), 5 were stage II, 2 were stage III, and 1 was unknown. No survival data are available for any of those patients.

## Discussion

Breast cancer treatment has evolved over the last 30 years. Patients diagnosed with early-stage disease are eligible for less morbid operations, with their adjuvant treatment largely dependent on stage and tumor biology^16–18^. Clinical trials, observational studies, and modeling studies have all demonstrated that regular screening mammography increases the likelihood of avoiding death from breast cancer^8^. Screening is endorsed by the National Comprehensive Cancer Network, US Preventative Task Force, and most major oncology societies^17–19^. Despite the known benefits of screening, many individuals face challenges due to limitations in access and/or prohibitive out-of-pocket expenses. By bringing free access to the community, the mammovan program provided screening to over 32,000 women in just 5 years and provided further assistance to women with abnormal results. Twelve percent of women (*n* = 3740) reported that their experience with the mammovan was their first screening event. Sixty-three percent of participants reported an annual income ≤$25,000, and 30% did not have health insurance at the time of screening.

Lower socioeconomic status has been connected to lack of adequate insurance coverage and, in the US, is strongly associated with race^1,20^. Data from the 2019 US Census Bureau demonstrate that 21% of African Americans live below the federal poverty level^21^. A similar pattern is evident in the rate of uninsured individuals, which approaches 15% among African Americans^22^. In our study, more than half of participants identified as either African American or Hispanic, two groups that appear to be disproportionally affected by disparities in health care outcomes^23–25^. The Bronx had the highest proportion of African American (30%) and Hispanic (37%) participants, the highest rate of annual income <$10,000 (21%), and the second highest rate of uninsured participants (31%). With the recognition of how race, socioeconomic status, and insurance status contribute to health care disparities, it should come as little surprise that the Bronx (190.2 average deaths among 931.8 annual cases) has the worst breast cancer–specific survival of the five boroughs^26^. The AICF program has taken the initiative to bridge the gap to access by bringing resources to these underserved communities, as more than one-quarter of the participants were found to reside in the two areas of New York City with the worst breast cancer outcomes.

Our data are consistent with what has been seen in the literature regarding the use of mobile screening mammograms (Table 4). A 2018 meta-analysis evaluated the ability of mobile mammography units to reach underrepresented populations, with the majority of participants identifying as African American or Latina, low income, and/or uninsured^27^. Similar to our study, the authors concluded that mobile mammography clinics may be effective at reaching medically underserved women, further adding that patient navigation to mobile mammography programs may promote greater attendance and increase follow-up adherence. The authors also speculated that some difficulties faced by mobile clinics were patient follow-up of abnormal or inconclusive findings^27^. These concerns are valid for many of programs with limited resources. In our study, more than 5000 women were assisted with additional testing, attesting to the robust program set in place by the AICF to ensure that women who were found to have an abnormal exam were able to undergo and follow through with the recommended follow-up.

**Table 4:** Current literature addressing mobile mammogram screening in the United States.

Reference (year)	Location (time frame)	No. of participants	Demographic information of participants	Breast cancers detected (%)
Brooks et al.^28^	Kentucky (2008–2010)	3923	48% African American 56% uninsured	31/3923 (0.0079)
McElfish et al.^33^	Arkansas (2010–2012)	5850	Rural, low education, and more likely to be non-Hispanic	–
Spak et al.^34^	Texas (2012–2017)	9327	76% Hispanic	14/9327 (0.0015)
Tsapatsaris et al.^35^	New York (2019)	3745	66% of participants identified as Hispanic and/or African American 43% uninsured 15% Medicare	17/3745 (0.005)

In 1995, Mandelblatt et al.^9^ identified mammographic capacity of a geographic area as a predictor of stage at presentation and showed that area-level socioeconomic status is related to late-stage disease at presentation among residents of New York State. In 2009, Elting et al.^10^ reported on the unequal distribution of screening facilities in Texas and found that the lack of an in-county screening facility was associated with late-stage diagnosis of breast cancer. These studies, in addition to our own, reinforce just how much the social environment affects outcomes and how hampered access to screening portends a worse prognosis. From 2014 to 2018, more than 2500 breast cancer–related deaths were reported in New York State, with a rate of 18.7 deaths per 100,000 persons (95% CI, 18.4–19.1)^26^. When evaluated by borough, the highest proportion of deaths was seen in the Bronx, and the lowest (56.8 annual deaths among 407.8 annual cases) was seen in Staten Island. Manhattan, Brooklyn, and Queens had rates of 19.8 (95% CI, 28.6–21.1), 20.2 (95% CI, 19.2–21.3), and 16.3 (95% CI, 15.3–17.2), respectively^26^. With 276,480 estimated new breast cancer cases projected for the US in the upcoming year^23^, early detection remains crucial. In 2013, Brooks et al.^28^ reported on 31 cases of breast cancer detected using a mobile screening program in Arkansas, concluding that the mobile outreach initiative successfully engaged many women who had not had a recent mammogram.

In New York State, which comprises a large and diverse population of nearly 10 million women^29^, living in areas of lower education and lower income is linked with increased odds of presenting with advanced-stage breast cancer^30^. New York State has taken aggressive action to improve breast cancer screening. In addition to extending facility hours, developing education programs that stress the importance of mammography, and eliminating cost-sharing, these interventions have been crucial to further incentivize women to undergo screening. Approximately 68% of women in New York State aged 50–74 years have reportedly undergone a screening mammogram—the goal, however, remains higher, at 81.1%^31^. By bringing free and convenient screening access to the community, the AICF has moved the state closer to this goal by enabling the screening of more than 32,000 women, the additional testing of 5359 women with abnormal screening results, and the detection of at least 68 new cases of breast cancer. Additionally, new technology can be adapted to the mammovan, which can provide state-of-the-art screening to those who otherwise may have limited access to care.

The breast cancer detection rate for our cohort was 68/32,350 (0.0021%), which is lower than the current benchmark of 5/1000 (0.005%)^32^. There are several factors that may account for this discrepancy, including underreporting or missing data. Approximately 12% of patients who were recommended for additional testing were unable to be contacted and/or lost to follow-up. We can only speculate that this could have resulted in potentially missed cancers. These patients may have also sought their breast care elsewhere and simply elected not to report this to the AICF.

Mobile health units are increasingly used to address barriers to mammography screening. These mammovan programs also assist in raising breast cancer awareness and can be used throughout the year, including on weekends and holidays. Maintaining appropriate equipment, being adaptable to new and validated evidence-based technology, having adequate resources, and working with a dedicated and efficient team are pivotal factors in establishing a successful mobile mammography program. Providing mobile mammography services in partnership with community organizations can be effective in increasing access and education and decreasing barriers to screening, particularly in higher-risk populations.

Our study was retrospective in nature, which may have led to selection bias. Data collection was performed using the AICF voluntary self-reported registry. With this comes the inherent limitations associated with any large data set, including the possibility of underreporting and misclassifying data. Because of the nature of the data set, we are unable to surmise which groups of individuals were the most likely to require additional follow-up or be diagnosed with breast cancer. Lastly, we do not have all data pertaining to stage of diagnosis or treatment follow-up for most of the participants identified as having cancer. Despite these limitations, we feel that our study lends further support to the concept of mobile screening programs within the large and diverse population of individuals residing in New York City. This experience supports that new technology may be adapted to mobile mammovans to provide state-of-the-art screening. These results warrant additional attention to the social determinants of health as a component of care.

Disparities in breast cancer care are multifactorial and composed of both biologic and socioeconomic components, chief among them being limited access to care. Together, these forces insidiously erect barriers to screening and compromise further treatment options. Ensuring adequate access to screening, particularly for minorities, populations living in socioeconomically disadvantaged areas, and individuals cared for in the public sector, remains an important step to improve outcomes in a more even distribution and across all races. Initiatives such as mobile, no-cost screening programs can help by providing medical services, educational tools, and financial resources to targeted populations known to face some of these unique challenges.

## Methods

### Data collection

This study was considered exempt by the Memorial Sloan Kettering Cancer Center institutional review board. Data were collected on participants who participated in the AICF Mobile, No-Cost Breast Cancer Screening Program from 1 January 2014 to 31 December 2019. These data were then submitted by the AICF and accepted for review. Adult women aged 40–79 years who had not had a mammogram in the last 12 months were invited to participate. Each patient underwent a clinical breast exam (CBE) by a nurse practitioner, followed by a screening mammogram. All patients who presented with an abnormal CBE also had an abnormal mammogram.

In 2012, AICF upgraded the analog mammography equipment to a digital unit GE XR Senographe Essential FFDM Mobile. As of 2021, this equipment remains in use. Images were read by a board-certified radiologist contracted by the AICF from Multi Diagnostic Services (MDS). All MDS radiologists reading AICF patient films have passed FDA inspection in compliance with the Mammography Quality Standards Act.

Demographic information was collected on a voluntary basis from individuals participating at each screening location via a 1-page questionnaire (Fig. 3). These data are compliant with the privacy requirements of the Health Insurance Portability and Accountability Act. Any patient who required further diagnostic workup beyond a screening mammogram (including further clinical breast exam, diagnostic mammogram, ultrasound, magnetic resonance imaging, and/or tissue biopsy) was referred to a stand-alone facility. The direct cost incurred by the AICF is ~$175 per patient screened; this includes the cost of a screening mammogram, clinical breast exam, breast health education, outreach, post-screening services, and personnel expenses. No charges were incurred by the patient for the use of this program.

Patients found to have abnormalities on screening were contacted by an MDS medical provider and informed of their results via a telephone call and letter. An AICF patient-navigator then assisted patients who required additional testing through the continuum of care, making appointments and providing referrals for supportive services. Women who did not have health insurance and who met the financial eligibility requirements were enrolled in the Cancer Services Program of New York State at the time of their screening. The program was administered by New York City borough–specific medical providers, who also provided the diagnostic testing. If diagnosed with breast cancer, these women could then enroll in the Medicaid Cancer Treatment Program, a New York State program that covers the cost of treatment. Women with health insurance were covered for both imaging and treatment, and additional financial assistance could be provided if needed. Once diagnosed with a cancer, the patient was transitioned to the institution of their choice. No additional follow-up information was available to the AICF unless the patient chose to contact the program with updates.

### Statistical analysis

Sociodemographic characteristics, by borough of residence, including age group, income level, race, and insurance status, were compared using the *χ*^2^ test. All statistical analyses were performed using SAS software (version 9.4, SAS Institute, Cary, NC).

### Reporting summary

Further information on research design is available in the Nature Research Reporting Summary linked to this article.

## Supplementary information

Reporting Summary

## Data Availability

The data that support the findings of this study are available from the corresponding author upon reasonable request.
