# Supplementary material for: Mobile mammography in New York City: analysis of 32,350 women utilizing a screening mammogram program
Source: NPJ Breast Cancer. 2022 Jan 21;8:14. doi: 10.1038/s41523-022-00381-6 (PMC8782895; doi:10.1038/s41523-022-00381-6)
Supplement: Supplementary file 1 — Reporting Summary [file 41523_2022_381_MOESM1_ESM.pdf]

nature portfolio

Corresponding author(s): Virgilio Sacchini

Last updated by author(s): Oct 8, 2021

Reporting Summary

Nature Portfolio wishes to improve the reproducibility of the work that we publish. This form provides structure for consistency and transparency in reporting. For further information on Nature Portfolio policies, see our Editorial Policies and the Editorial Policy Checklist.

Statistics

For all statistical analyses, confirm that the following items are present in the figure legend, table legend, main text, or Methods section.

n/a	Confirmed	
☐	☒	The exact sample size (n) for each experimental group/condition, given as a discrete number and unit of measurement
☒	☐	A statement on whether measurements were taken from distinct samples or whether the same sample was measured repeatedly
☐	☒	The statistical test(s) used AND whether they are one- or two-sided<br>*Only common tests should be described solely by name; describe more complex techniques in the Methods section.*
☒	☐	A description of all covariates tested
☒	☐	A description of any assumptions or corrections, such as tests of normality and adjustment for multiple comparisons
☐	☒	A full description of the statistical parameters including central tendency (e.g. means) or other basic estimates (e.g. regression coefficient) AND variation (e.g. standard deviation) or associated estimates of uncertainty (e.g. confidence intervals)
☒	☐	For null hypothesis testing, the test statistic (e.g. F, t, r) with confidence intervals, effect sizes, degrees of freedom and P value noted<br>*Give P values as exact values whenever suitable.*
☒	☐	For Bayesian analysis, information on the choice of priors and Markov chain Monte Carlo settings
☒	☐	For hierarchical and complex designs, identification of the appropriate level for tests and full reporting of outcomes
☒	☐	Estimates of effect sizes (e.g. Cohen's d, Pearson's r), indicating how they were calculated

Our web collection on statistics for biologists contains articles on many of the points above.

Software and code

Policy information about availability of computer code

Data collection	Data were collected on participants who participated in the American Italian Cancer Foundation (AICF) Mobile, No-Cost Breast Cancer Screening Program from January 1, 2014, to December 31, 2019. These data were then submitted by the AICF and accepted for review.
Data analysis	Sociodemographic characteristics, by borough of residence, including age group, income level, race, and insurance status, were compared using the χ2 test. All statistical analyses were performed using SAS software (version 9.4, SAS Institute, Cary, NC).

For manuscripts utilizing custom algorithms or software that are central to the research but not yet described in published literature, software must be made available to editors and reviewers. We strongly encourage code deposition in a community repository (e.g. GitHub). See the Nature Portfolio guidelines for submitting code & software for further information.

Data

Policy information about availability of data

All manuscripts must include a data availability statement. This statement should provide the following information, where applicable:

- Accession codes, unique identifiers, or web links for publicly available datasets
- A description of any restrictions on data availability
- For clinical datasets or third party data, please ensure that the statement adheres to our policy

The data that support the findings of this study are available from the corresponding author upon reasonable request.

Field-specific reporting

Please select the one below that is the best fit for your research. If you are not sure, read the appropriate sections before making your selection.

- [ ] Life sciences
- [x] Behavioural & social sciences
- [ ] Ecological, evolutionary & environmental sciences

For a reference copy of the document with all sections, see nature.com/documents/nr-reporting-summary-flat.pdf

Behavioural & social sciences study design

All studies must disclose on these points even when the disclosure is negative.

Study description	Retrospective Review
Research sample	Adult women living in New York City, aged 40 to 79 years, who had not had a mammogram in the last 12 months were invited to participate. Each patient underwent a clinical breast exam (CBE) by a nurse practitioner, followed by a screening mammogram. All patients that had an abnormal CBE also had an abnormal mammogram.
Sampling strategy	Adult women living in New York City, aged 40 to 79 years, who had not had a mammogram in the last 12 months were invited to participate.
Data collection	Demographic information was collected on a voluntary basis from individuals participating at each screening location via a 1-page questionnaire. These data are compliant with the privacy requirements of the Health Insurance Portability and Accountability Act.
Timing	Jan 2014-Dec 2019
Data exclusions	Incomplete data were exluded
Non-participation	NA
Randomization	NA

Reporting for specific materials, systems and methods

We require information from authors about some types of materials, experimental systems and methods used in many studies. Here, indicate whether each material, system or method listed is relevant to your study. If you are not sure if a list item applies to your research, read the appropriate section before selecting a response.

Materials & experimental systems

n/a	Involved in the study	
☒	☐	Antibodies
☒	☐	Eukaryotic cell lines
☒	☐	Palaeontology and archaeology
☒	☐	Animals and other organisms
☒	☐	Human research participants
☒	☐	Clinical data
☒	☐	Dual use research of concern

Methods

n/a	Involved in the study	
☒	☐	ChIP-seq
☒	☐	Flow cytometry
☒	☐	MRI-based neuroimaging
